# *Best of* du 2^e^ congrès de la Société sénégalaise de pathologie infectieuse et tropicale (SOSEPIT), Dakar 28 au 30 avril 2025: Organisateur : Société sénégalaise de pathologie infectieuse et tropicale (SOSEPIT)

**DOI:** 10.48327/mtsi.v6i1.2026.801

**Published:** 2026-01-23

**Authors:** Aminata MASSALY, Agbogbenkou Tevi Dela Dem LAWSON, Moustapha DIOP, Maimouna SIDIBE, Aboubacar Sadikh BADIANE, Ndeye Maguette FALL, Daouda THIOUB, Kourro BOUSSO, Aminata THIAM, Alassane DIEYE, Kalilou DIALLO, Viviane Marie Pierre CISSE, Daye KA, Ndeye Aissatou LAKHE, Khardiata DIALLO-MBAYE, Pape Samba BA, Ndèye Fatou Ngom GUEYE, Louise FORTES, Noël Magloire MANGA, Sylvie Audrey DIOP, Khadidiatou Maïmouna BA FALL, Moussa SEYDI, Loïc EPELBOIN, Ndeye Mery DIA BADIANE

**Affiliations:** 1 Service des maladies infectieuses et tropicales, Centre hospitalier universitaire Fann, Dakar, Sénégal, Université Cheikh Anta Diop Dakar (UCAD), Sénégal; 2 Unité de formation et de recherche en sciences de la santé, Université Iba Der Thiam (UIDT) de Thiès, Sénégal; 3 Services des maladies infectieuses, Hôpital principal de Dakar, Sénégal; 4 Division de lutte contre le SIDA et les IST (DLSI), ministère de la Santé et de l’action sociale, Sénégal; 5 Directrice technique du projetd EpiC FHI360, Sénégal; 6 Unité de formation et de recherche en sciences de la santé, Université Gaston Berger, (UGB), Sénégal; 7 Unité de formation et de recherche en sciences de la santé, Université Assane Seck de Ziguinchor (UASZ), Sénégal; 8 Présidente du Comité d’organisation du 2^e^ congrès de la Société sénégalaise de pathologie infectieuse et tropicale, Unité de formation et de recherche en sciences de la santé, Université Alioune Diop de Bambey, Sénégal; 9 Présidente du Comité scientifique du 2^e^ congrès de la Société sénégalaise de pathologie infectieuse et tropicale, Université Cheikh Anta Diop Dakar, Sénégal; 10 Trésorière générale de la Société sénégalaise de pathologie infectieuse et tropicale; 11 Unité des maladies infectieuses et tropicales, CIC Inserm 1424, UA17 Santé des populations amazoniennes, Université de Guyane - CHU de Guyane- Cayenne 97300, France; 12 Présidente de la Société sénégalaise de pathologie infectieuse et tropicale, UFR des sciences de la santé, Université Gaston Berger, Service de médecine, Centre hospitalier régional de Saint-Louis, Sénégal

## RÉSUMÉ DU CONGRÈS ET DU PRÉCONGRÈS

### Pré-congrès, CHU Fann, Dakar, 28 avril 2025 Thème : Prévention des infections associées aux soins (IAS)

La journée précongrès du 2^e^ congrès de la SOSEPIT a été consacrée à la prévention des infections associées aux soins, enjeu majeur de sécurité des patients en Afrique. Après l’accueil des participants et l’ouverture officielle par les organisateurs, les échanges ont débuté par une session introductive sur les concepts clés des IAS, animée par les Prs Louise Fortes et Aissatou Lakhe. Cette session a été suivie d’un atelier pratique sur l’hygiène des mains et le port de gants, illustrant les gestes barrières essentiels.

La matinée s’est poursuivie avec un second atelier sur les précautions complémentaires (contact, air, gouttelettes), présenté par les Drs Sokhna Daffe et Tracie Youbong, axé sur l’adaptation des mesures préventives aux risques spécifiques. Un troisième atelier, animé par le Pr Daye Ka et Mme Aïssa Sow Barry, a abordé la prévention des IAS liées aux dispositifs médicaux, avec un focus sur les bonnes pratiques de pose, d’entretien et de retrait.

La clôture du précongrès a permis de synthétiser les messages clés et de remettre les certificats de participation à 169 agents de santé. Cette journée interactive, combinant apports théoriques et démonstrations pratiques, a offert aux participants un socle commun pour renforcer la lutte contre les IAS dans leurs structures respectives.

### Congrès, Hôtel Azalaï, Dakar, 28-30 avril 2025

Le 2^e^ congrès de la Société sénégalaise de pathologie infectieuse et tropicale (SOSEPIT) s’est tenu à Dakar du 28 au 30 avril 2025. Rassemblant un large éventail de professionnels de santé, chercheurs, enseignants, autorités sanitaires et partenaires internationaux de l’Afrique francophone (Burkina Faso, Congo, Côte d’Ivoire, Gabon, Guinée, Mali, Niger et République démocratique du Congo) mais aussi de la Guyane, cet événement scientifique a offert une plateforme d’échanges sur les enjeux actuels et futurs en infectiologie, avec une attention particulière portée à la résistance aux antimicrobiens (RAM), aux maladies émergentes, aux approches préventives et aux innovations diagnostiques et thérapeutiques adaptées aux réalités africaines. Les conférences plénières ont posé les bases des réflexions stratégiques. Le Pr Noël Manga a introduit le concept d’approche « *One Health* » comme levier central dans la lutte contre la RAM, soulignant l’importance d’une action coordonnée entre médecine humaine, santé animale et écologie. Le Pr Mandicou Ba a poursuivi avec une intervention sur les apports actuels et futurs de l’intelligence artificielle en infectiologie, en particulier dans les domaines du dépistage précoce, du diagnostic automatisé et de l’analyse prédictive appliquée aux épidémies. Le Pr Loïc Epelboin a présenté un panorama des émergences infectieuses dans le monde sur la période 2024-2025, incluant les épidémies de fièvres hémorragiques virales, les résurgences de dengue et chikungunya et les risques liés aux zoonoses en zones tropicales. Par ailleurs, le Dr Ibrahima Diouf a exploré les liens croissants entre changement climatique et dynamique des maladies infectieuses, insistant sur l’impact des dérèglements environnementaux sur la répartition géographique des vecteurs et des pathogènes. Deux autres conférences plénières ont mis en lumière des enjeux plus ciblés : le Pr Bécaye Fall a évoqué les défis structurels de la surveillance de la résistance aux antibiotiques en Afrique, tandis que le Pr Halimatou Diop Ndiaye a abordé la problématique émergente de la résistance au dolutégravir chez les personnes vivant avec le VIH.

Les symposiums ont permis d’approfondir les pratiques cliniques, en particulier autour de l’antibiothérapie dans le contexte des infections à bactéries multirésistantes. Des recommandations actualisées ont été discutées à partir de l’expérience des praticiens de terrain, avec un focus sur l’optimisation de l’usage des carba-pénémes, les alternatives thérapeutiques accessibles en Afrique, et la nécessité d’une meilleure régulation de la prescription. Un second symposium a été consacré aux maladies tropicales négligées (MTN), encore trop peu prises en compte dans les politiques de santé malgré leur forte prévalence en Afrique subsaharienne. Les sessions parallèles ont couvert un large spectre de pathologies infectieuses et de problématiques de santé publique. Certaines étaient centrées sur des agents infectieux majeurs tels que le VIH, les hépatites virales et la tuberculose, tandis que d’autres ont exploré les maladies fongiques invasives, les infections parasitaires comme le paludisme, ainsi que les maladies tropicales négligées. Une session spécifique a été consacrée aux liens entre maladies non transmissibles (diabète, cancer, etc.) et infections opportunistes, montrant l’importance croissante de ces interactions dans les contextes africains.

Parmi les moments forts du congrès, la conférence inaugurale prononcée par le Pr Khadidiatou M. Ba Fall a souligné que la résistance aux antimicrobiens constitue l’un des défis sanitaires les plus urgents pour le continent africain, appelant à une mobilisation collective des institutions, des professionnels et de la société civile. Les tables rondes ont favorisé les échanges d’expériences et les débats sur des questions pratiques. L’une d’elles a porté sur les difficultés diagnostiques des infections fongiques en Afrique, en lien avec l’indisponibilité des tests spécifiques, le retard au diagnostic et l’accès limité aux antifongiques de référence. Une autre table ronde a réuni des experts du ministère de la Santé, de la SOSEPIT et des structures de santé, pour discuter de la surveillance des fièvres hémorragiques virales (FHV) au Sénégal, en insistant sur l’importance d’un renforcement des dispositifs de détection précoce et de réponse rapide.

Enfin, les sessions sur les maladies émergentes, les approches « *One Health* » et la gestion des infections dans les contextes de fragilité ont permis d’aborder les perspectives futures de la lutte contre les épidémies. Ces échanges ont mis en évidence le besoin d’intégration entre les disciplines, d’harmonisation des protocoles et d’investissement dans la formation et la recherche appliquée.

Ce congrès a ainsi confirmé le dynamisme croissant de la communauté infectiologique en Afrique de l’Ouest, son engagement dans une approche rigoureuse de santé publique et la volonté collective de renforcer la souveraineté sanitaire dans la région. Il s’est clôturé sur des perspectives encourageantes de coopération scientifique, de mutualisation des ressources et d’innovation au service des populations.

Est proposé ici un *best of* des présentations du congrès de la SOSEPIT, avec la partialité d’un invité extérieur féru de médecine tropicale et de zoonoses, lesquelles n’étaient pas les thématiques principales du congrès.

**Figure 1:**
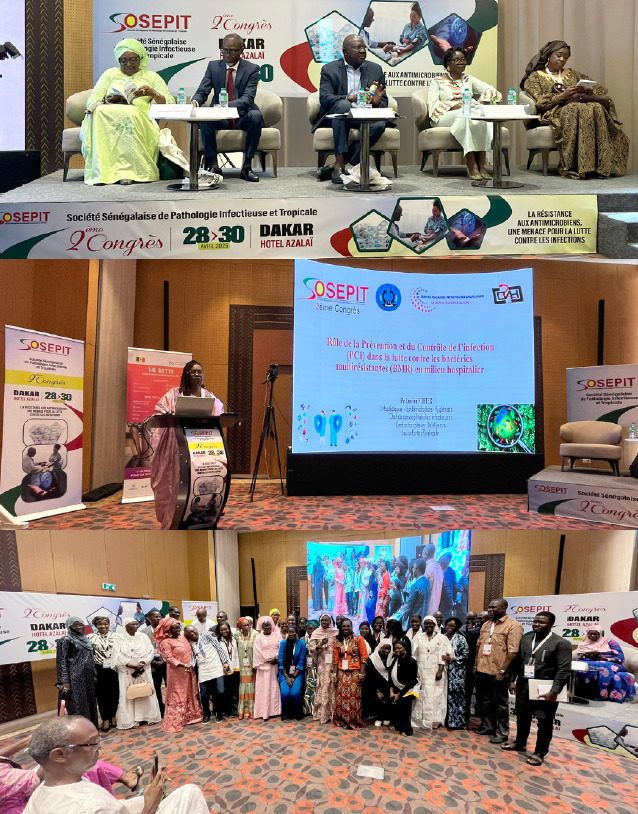
Photos prises lors du 2^e^ congrès de la Société sénégalaise de pathologie infectieuse et tropicale (SOSEPIT), Dakar 28 au 30 avril 2025 (crédit photo : L. Epelboin)

