# Supplementary material for: Highlights from the 2nd Congress of the Senegalese Society of Infectious and Tropical Pathology (SOSEPIT), Dakar, April 28–30, 2025
Source: Med Trop Sante Int. 2026 Jan 23;6(1):mtsi.v6i1.2026.801. [Article in French] doi: 10.48327/mtsi.v6i1.2026.801 (PMC13216920; doi:10.48327/mtsi.v6i1.2026.801)
Supplement: Related texts

# Research article: Prévalence des bactéries multirésistantes en Afrique de l’Ouest : revue systématique avec méta-analyse.

**Published:** 2026-01-23

**Authors:** Moustapha DIOP

**Affiliations:** Hôpital principal de Dakar, Sénégal

La résistance bactérienne aux antibiotiques est un enjeu de santé publique majeur, en particulier en Afrique de l’Ouest où elle est responsable d’un taux élevé de morbidité et de mortalité, estimé à 27,3 décès pour 100 000 habitants en 2019. Cette revue systématique avec méta-analyse visait à estimer la prévalence globale des bactéries multirésistantes (BMR) dans les 16 pays d’Afrique de l’Ouest, en suivant les standards PRISMA de 2010 à 2024.

Les bases de données consultées incluaient PubMed/MEDLINE, CINAHL, Google Scholar, African Index Medicus, AJOL et la littérature grise. Cinquante études ont été retenues (dont 17 (34 %) au Nigéria et 11 (22 %) au Ghana), 3 pays n’ayant pas de données disponibles (Mauritanie, Guinée et Cap-Vert), portant sur 35 516 isolats bactériens, dont 20 495 étaient multirésistants (définis par une résistance à au moins trois classes d’antibiotiques).

Les résultats montrent une prévalence globale des BMR de 59 % [IC95 % : 48-69 %], avec une forte hétérogénéité inter-études (I² = 98 %). Les bacilles à Gram négatif représentaient 83,9 % des souches isolées, avec une prédominance de *Escherichia coli* et *Klebsiella pneumoniae*. L’analyse en sous-groupes n’a pas mis en évidence de différence significative entre les infections communautaires et nosocomiales. Toutefois, la prédominance des infections urinaires était marquée.

Cette revue systématique a révélé une forte prévalence des BMR dans les établissements de santé et les communautés d’Afrique de l’Ouest. Bien que les BMR soient plus fréquemment observées dans les infections nosocomiales, leur forte prévalence au niveau communautaire est également préoccupante. Ces résultats soulignent l’importance de renforcer les pratiques de prévention et de contrôle des infections dans les hôpitaux et la nécessité d’une surveillance plus rigoureuse des BMR en Afrique de l’Ouest. Des politiques strictes en matière de consommation et d’utilisation des antibiotiques sont également nécessaires pour prévenir la propagation des bactéries multirésistantes. En outre, l’utilisation d’une définition standardisée des bactéries multirésistantes serait utile pour estimer plus précisément leur prévalence en Afrique de l’Ouest.

# Research article: Évolution des profils de résistance d’*Escherichia coli* à l’Hôpital principal de Dakar, Sénégal

**Published:** 2026-01-23

**Authors:** Maguette NDOYE

**Affiliations:** Biologie, Hôpital principal de Dakar, Sénégal Prix de la meilleure communication orale

Cette étude rétrospective menée sur 10 ans (2012-2021) au laboratoire de microbiologie de l’Hôpital principal de Dakar visait à analyser l’évolution de la résistance aux antibiotiques chez *Escherichia coli*, bactérie commensale impliquée dans de nombreuses infections courantes (infections urinaires, méningites néonatales, bactériémies, etc.). Sur plus de 23 000 antibiogrammes réalisés, 7 797 souches d’*E. coli* ont été isolées (33,4 %). L’âge médian des patients était de 52 ans (IQR 23-69), avec une légère prédominance masculine (sex-ratio H/F 1,1). Les résultats montrent une résistance globale sur la période d’étude de 85 % pour l’ampicilline et la ticarcilline, 68 % pour le cotrimoxazole, 57 % pour l’acide nalidixique, 43 % pour la pefloxacine, 28 % pour la gentamicine et 27 % au cefotaxime et à la ceftazidime, avec 24 % de souches productrices de BLSE. On constate une augmentation constante des taux de résistance entre 2012 et 2021 aux céphalosporines de troisième génération (C3G) (28 % à 41 %), à l’amoxicilline (38 % à 65 %), ou à l’imipénème (0,1 à 3,2 %). La discussion a souligné la facilité de dissémination d’*E. coli* dans les milieux hospitaliers et communautaires *via* les plasmides, ainsi que la nécessité de renforcer la politique de bon usage des antibiotiques et d’investir dans la biologie moléculaire pour suivre les mécanismes de résistance. Ce travail met en lumière l’ampleur silencieuse de la multirésistance d’*E. coli* dans un hôpital sénégalais de référence.

# Research article: Résistance aux antibiotiques chez *Acinetobacter baumannii* à Bamako, Mali

**Published:** 2026-01-23

**Authors:** Abdoulaye Mamadou TRAORE

**Affiliations:** Faculté de médecine de Bamako, Université des sciences, des techniques et de technologie de Bamako, Mali

Présentée comme une des bactéries les plus problématiques dans la lutte contre la RAM, *Acinetobacter baumanii* fait partie de la liste prioritaire de l’OMS. Ce travail, mené à Bamako à partir de prélèvements hospitaliers et communautaires, a porté sur 19 souches isolées entre 2018 et 2019 parmi 583 bactéries étudiées, soit 3,6 %. Cette entérobactérie était retrouvée dans 58 % des cas dans des expectorations. Les résultats montrent des niveaux de résistance élevés à de nombreux antibiotiques (ticarcilline, ticarcilline et acide clavulanique : 53%; pipéracilline-tazobactam et ceftazidime : 47 %; céfépime : 47%; imipénème et méropénème : 32 %). Le gène de carbapénémase blaOXA-23 était présent chez 83 % des souches testées, associé à l’élément d’insertion ISAbA1 dans 100 % des cas. La colistine reste active (0 % de résistance), mais son usage doit être réservé en dernier recours, d’autant qu’elle est difficilement accessible dans la plupart des hôpitaux d’Afrique de l’Ouest. Ce travail souligne la progression rapide de la résistance d’*A. baumanii* dans les hôpitaux maliens et l’urgence de mettre en place une surveillance continue, couplée à des mesures strictes d’hygiène et à une rationalisation de la prescription antibiotique.

# Research article: Résistance aux antibiotiques chez *Pseudomonas aeruginosa* à Bamako, Mali

**Published:** 2026-01-23

**Authors:** Abdoulaye Mamadou TRAORE

**Affiliations:** Faculté de médecine de Bamako, Université des sciences, des techniques et de technologie de Bamako, Mali

*Pseudomonas aeruginosa*, bacille opportuniste, est responsable d’infections nosocomiales sévères, notamment chez les patients immunodéprimés. Cette étude transversale menée à Bamako sur 13 mois de janvier 2020 à avril 2021 sur divers échantillons a permis d’isoler 56 souches de *P. aeruginosa* sur 583 prélèvements soit 9,6 %. La majorité des souches était hospitalière (82 %), provenant d’expectorations, urines, pus ou liquides de ponction. Les taux de résistance aux bêtalactamines et aux aminosides étaient élevés (ticarcilline 46 %, pipéracilline tazobactam 34 %, ceftazidime et céfépime 23 %, imipénème 11 %, amikacine 20 %, gentamicine 27 %, ciprofloxacine 21 %), avec un profil multirésistant dans 9 % des cas. Une analyse génotypique par PCR a révélé la présence du gène VIM-2 chez les 5 souches résistantes aux carbapénémes, toutes isolées en milieu hospitalier. La comparaison avec des données antérieures montre une persistance des taux élevés de résistance de *P. aeruginosa* aux antibiotiques de 1^re^ ligne. L’étude souligne la nécessité de renforcer la surveillance moléculaire et d’améliorer l’hygiène hospitalière pour limiter la transmission croisée de ces souches résistantes.

# Research article: Émergence des carbapénémases dans les hôpitaux du Niger

**Published:** 2026-01-23

**Authors:** Mahamadou DOUTCHI

**Affiliations:** Faculté des sciences de la santé de l’Université André Sali-fou, et Hôpital national de Zinder, Niger

L’émergence des carbapénémases constitue une menace majeure de santé publique. Elle pourrait être à l’origine d’une augmentation de la mortalité du fait de la limitation des options thérapeutiques qu’elle engendre. L’objectif de cette étude était de déterminer la circulation de carbapénémases dans deux hôpitaux du Niger. Il s’agissait d’une étude descriptive transversale portant sur les souches bactériennes isolées dans divers prélèvements aux laboratoires de biologie médicale de l’hôpital national de Zinder et de l’hôpital de référence de Maradi du 1^er^ août 2022 au 31 juillet 2023. Les souches étaient ensemencées sur les géloses ChromID® carba et ChromID® BLSE pour l›isolement des bactéries productrices de BLSE et des carbapénémases. L’identification des souches était faite par MAL-DI-TOF/MS. Au total, 123 souches bactériennes étaient identifiées au MALDI-TOF/MS parmi lesquelles la prévalence des BMR était de 74 %. La fréquence de BMR était plus élevée pour l’espèce *Escherichia coli* (60,44 %) suivie du *Klebsiella pneumoniae* (20,88 %). Ces BMR étaient isolées majoritairement dans les urines (63,74 %). Le phénotype de résistance BLSE était le plus fréquent (76,25 %). Toutes les souches productrices de BLSE présentaient une résistance associée aux fluoroquinolones. Les gènes de BLSE détectés étaient constitués de TEM-1, TEM-2, CTXM-1 like et OXA-1 like. La résistance des souches aux carbapénémes était retrouvée dans 6,59 % des cas. Pour ces carbapénémases les gènes de résistance retrouvés étaient des NDM et une suspicion d’OXA-48 like. Cette étude met en évidence une émergence des carbapénémases dans nos régions. Il importe de respecter les mesures de prévention et de contrôle des infections (PCI) et de renforcer l’utilisation rationnelle des antibiotiques. Des études à l’échelle nationale seraient nécessaires pour compléter cette étude.

# Research article: Expérience de l’Équipe mobile d’antibiothérapie (EMA) de l’Hôpital principal de Dakar

**Published:** 2026-01-23

**Authors:** Sokhna Moumi Mbacké DAFFE

**Affiliations:** Hôpital principal de Dakar, Sénégal

L’Hôpital principal de Dakar, hôpital d’instruction des armées d’une capacité de 365 lits, s’est doté en août 2023 de la première Équipe mobile d’antibiothérapie (EMA) d’Afrique francophone, une structure innovante et multidisciplinaire ayant pour mission de rationaliser la prescription des antibiotiques. L’équipe est constituée d’un médecin chef chapeautant 2 coordinateurs, l’un infectiologue et l’autre biologiste, eux-mêmes coordonnant une équipe de 3 infectiologues, 2 biologistes, 2 pharmaciens et 2 hygiénistes en contact avec les référents de 20 services différents. L’EMA a pour missions de former les professionnels de santé, sensibiliser les équipes à la lutte contre l’antibiorésistance, surveiller l’usage des antibiotiques, conseiller les prescripteurs, évaluer les pratiques et optimiser les traitements antiinfectieux au sein des établissements de soins. Les mésusages les plus fréquents concernaient l’antibioprophylaxie chirurgicale, la confusion entre colonisation et infection des voies urinaires, les antibiothérapies à tort des infections superficielles du site opératoire, et une surprescription des aminosides. En 18 mois, l’équipe a réalisé des consultations pour plus de 520 patients (18 % de signalements d’hémocultures et 82 % de sollicitations d’avis), avec un taux d’intervention avant antibiothérapie de 36 % (abstention 8 %, initiation 24 %) et une réadaptation des antibiotiques dans 47 % des cas (changement d’antibiotique : 36 %, diminution du nombre d’antibiotiques : 14 %, etc.). L’EMA a également mené des formations ciblées envers les médecins et les paramédicaux, participé à la rédaction d’un livret d’antibiothérapie personnalisé à l’hôpital et organisé un séminaire d’antibiothérapie en avril 2024. Ce bilan exemplaire illustre le potentiel d’un modèle transférable à d’autres hôpitaux africains, et montre que l’implémentation locale de bonnes pratiques peut avoir un impact significatif sur la lutte contre la résistance.

# Research article: Bactériémies associées aux soins en néonatologie à l’hôpital Dalal Jamm

**Published:** 2026-01-23

**Authors:** Ndèye Fatou SOW

**Affiliations:** Service d’hygiène hospitalière, hôpital Dalal Jamm, Dakar, Sénégal - Prix de la meilleure communication orale

Les bactériémies sont la principale infection nosocomiale en néonatologie. Cette étude se fixait pour objectifs de déterminer l’incidence des bactériémies associées aux soins à l’unité de néonatologie de l’hôpital Dalal Jamm au Sénégal, d’évaluer le dispositif de prévention du risque infectieux (PRI) et de mettre en œuvre des actions afin de renforcer la prévention. Une étude longitudinale rétrospective a été réalisée d’octobre 2022 à juillet 2023. Ont été inclus tous les nouveau-nés présentant une hémoculture positive après un délai de 48 heures après l’admission. Les données ont été recueillies à partir du registre de laboratoire et des dossiers de patients. Afin d’évaluer le dispositif de prévention du risque infectieux (PRI) en néonatologie, des observations ont été réalisées et le personnel soignant a été interrogé à l’aide d’une grille. Le niveau de connaissance des soignants sur l’hygiène des mains a été évalué par auto-administration. Au total, 36 cas ont été inclus sur 288 patients admis soit une incidence cumulée de 12,5 % et une densité d’incidence de 15,5 cas/1 000 patients–jour. Un pic a été noté en février 2023 avec une densité d’incidence de 43,5 cas/1 000 patients–jour. Le délai médian de survenue de l’IAS était de 6 jours [2-13]. Treize sont décédés, soit une létalité hospitalière de 36 %. Les germes isolés étaient des bacilles Gram négatif dans deux tiers des cas (65,8 %), dominés par les entérobactéries (47,3 %) : *Enterobacter, Klebsiella et Escherichia*. En dehors de la gestion des excrétas et des parents, les niveaux de performances pour la PRI étaient globalement inférieurs à 50 % dans les différentes rubriques. Globalement, on a noté une bonne connaissance des 5 indications de l’hygiène des mains. Toutefois, 70 % des enquêtés pensaient que la friction hydro-alcoolique (FHA) était moins efficace que le lavage des mains et 80 % que les FHA n’agissaient pas sur les bactéries multirésistantes (BMR). Vingt-et-un pour cent des soignants ont affirmé qu’il fallait réaliser un lavage des mains avant toute FHA. En conclusion, l’incidence des bactériémies a évolué au cours des mois avec une épidémie au mois de février 2023. Le niveau de performance pour la PRI est globalement faible, de même que les connaissances sur l’efficacité de la FHA. Les actions entreprises doivent être maintenues et renforcées afin de prévenir la survenue des IAS en néonatologie.

# Research article: Résultats du traitement de la tuberculose résistante à la rifampicine en Afrique subsaharienne : étude multicentrique DIAMA

**Published:** 2026-01-23

**Authors:** Awa BA DIALLO

**Affiliations:** Laboratoire de bactériologie-virologie, hôpital Dalal Jamm, Dakar, Sénégal

La tuberculose résistante à la rifampicine (TBRR) pose des défis importants au contrôle de la tuberculose, y compris en Afrique subsaharienne. L’optimisation des soins et la prévention de la TB-RR nécessitent une compréhension approfondie des principaux facteurs associés à l’échec du traitement. Nous avons évalué les résultats des programmes de traitement des patients atteints de TB-RR dans plusieurs pays africains. Il s’agissait d’une étude prospective multicentrique à travers neuf pays d’Afrique subsaharienne (Bénin, Éthiopie, Cameroun, Mali, RDC, Rwanda, Guinée, Nigeria et Sénégal) entre juillet 2017 et mars 2021. Les patients consentants âgés de 15 ans et plus étaient recrutés sur la base des résultats Xpert MTB/RIF révélant une TB-RR, et avaient reçu un traitement contre la TB-RR conformément aux directives nationales, généralement le régime de traitement court de 9 mois contenant des injectables, ou un régime de 18 à 21 mois, avec un suivi pendant 6 mois après le traitement. Un traitement réussi comprenait la guérison et l’achèvement complet du traitement, tandis que le décès, les perdus de vue et l’échec étaient considérés comme des traitements non réussis. Les facteurs de risque ont été évalués à l’aide de la régression logistique. Sur les 1 906 patients TB-RR recrutés, près de 1 845 (97 %) avaient été traités avec le régime court, 1 315 (69 %) étaient des hommes et l’âge moyen était de 35 ans. Le suivi médian après traitement était de 4 mois. Au total, 1 373 (96 %) patients avaient obtenu un traitement réussi, avec 985 (54 %) qui avaient été guéris et 388 (21 %) ayant un traitement complet. Parmi les 455 patients restants, 217 (11,9 % du total) étaient décédés, 29 (1,6 %) avaient échoué et 209 (11,4 %) avaient été perdus de vue. Le taux de réussite du traitement variait d’un pays à l’autre, allant de 61 % au Nigeria à 90 % au Rwanda. Les facteurs significativement associés à l’échec du traitement comprenaient les résultats de la culture de base avec un OR ajusté (ORa) (ORa=0,79; 95 %CI : 0,63-0,99), le régime de traitement (ORa=0,50; 95 %CI : 0,40-0,61), et les résultats Xpert MTB/ RIF (ORa=0,70; 95 %CI : 0,48-1,02). Le taux de réussite du traitement était inférieur à l’objectif de 75 % fixé par l’OMS pour les participants à l’étude au Nigeria, en Guinée, au Mali et au Sénégal, mais il dépassait largement l’objectif fixé au Bénin, au Rwanda, en Éthiopie, en RDC et au Cameroun. L’étude recommande de renforcer les programmes de lutte contre la tuberculose et d’évaluer les régimes de traitement en tenant compte de facteurs spécifiques à chaque pays.

# Research article: Leishmaniose cutanée : origine géographique et spectre clinique chez 62 patients

**Published:** 2026-01-23

**Authors:** Assane DIOP

**Affiliations:** Dermatologie, IST Hôpital Institut d’hygiène sociale de Dakar, Sénégal

Cette étude rétrospective sur 11 ans (2014-2024) a analysé 62 cas de leishmaniose cutanée diagnostiqués dans le Service de dermatologie de l’hôpital Institut d’hygiène sociale de Dakar. Seuls 25 % avaient des comorbidités (principalement HTA). Bien que cette parasitose soit habituellement décrite en zones rurales ou sahéliennes, l’étude montre une urbanisation croissante des cas : 53 % des patients résidaient à Dakar, dont la moitié sans notion de séjour hors de la capitale. L’âge moyen était de 32 ans, le sex-ratio proche de 1, la durée moyenne d’évolution de la lésion avant diagnostic de 3,5 mois. Le spectre clinique était varié, avec une prédominance de la forme ulcéro-croûteuse (89 %), suivie de la forme pseudo-sporotrichosique (21 %), puis ulcéro-surinfectée (13 %). Les membres supérieurs étaient atteints dans 79 % des cas, les membres inférieurs dans 36 % des cas et la tête dans 27 % des cas. La comparaison dans la littérature montre que la proportion des patients strictement dakarois passe de 2 % dans une publication de 2016 à 13 % dans une publication de 2018 puis à 26 % dans cette étude. Cette diversité clinique et la progression géographique de la maladie interpellent : l’urbanisation rapide, les grands travaux d’aménagement et la désertification sont suspectés d’avoir modifié la dynamique éco-épidémiologique. Ce travail met en lumière une réalité sous-estimée de la leishmaniose à Dakar et l’importance de l’inclure dans le diagnostic différentiel des lésions cutanées chroniques en milieu urbain.

# Research article: Intelligence artificielle pour le diagnostic des maladies tropicales négligées (MTN) de la peau au Sénégal et en Afrique de l’Ouest

**Published:** 2026-01-23

**Authors:** Pauline DIOUSSE NGOM

**Affiliations:** Unité de formation et de recherche en sciences de la santé, Université Iba Der Thiam (UIDT) de Thiès, Sénégal

Cette présentation innovante a rendu compte d’une étude pilote, le projet DAHW (Deutsche Lepra- und Tuberkulosehilfe e.V), évaluant l’intérêt et la faisabilité d’une application de smart-phone d’intelligence artificielle utilisée pour le dépistage de maladies tropicales négligées à manifestation cutanée (lèpre, gale, mycétome, leishmaniose, filariose lymphatique…). Sur 814 images dermatologiques recueillies dans trois sites (Thiès, Dakar et Mballing), 111 (13 %) ont été identifiées comme MTN, avec une bonne spécificité du diagnostic (97 %). Toutefois, des limites techniques ont été relevées : qualité variable des images, mauvaise performance sur les peaux foncées, et manque de formation des modèles sur les populations africaines. Le projet illustre néanmoins le potentiel des outils numériques pour améliorer la détection précoce au niveau communautaire. Des perspectives de développement sont en cours, incluant des projets de recherche sur les algorithmes adaptés aux peaux noires. Cette initiative s’inscrit dans une dynamique de modernisation et d’approche intégrée de la lutte contre les MTN à l’interface santé et technologie.

# Research article: Aspects actuels de la dengue au service des maladies infectieuses du Centre hospitalier universitaire du Point G (Bamako)

**Published:** 2026-01-23

**Authors:** Yacouba CISSOKO

**Affiliations:** Université des sciences, techniques et technologies de Bamako, CHU du Point G, Mali

Dans un contexte de flambée régionale de dengue survenue à partir de septembre 2023, cette étude prospective conduite au CHU du Point G à Bamako entre septembre 2024 et janvier 2025 apporte des données actualisées sur l’épidémiologie et la gravité de cet arbovirus dans un centre de référence. Ainsi, 347 patients atteints de dengue confirmée ont été inclus, par RT-PCR, antigène NS1 ou IgM. Parmi eux, 205 ont été suivis en ambulatoire et 142 hospitalisés, soit un taux d’hospitalisation de 40,9 %.

Les patients étaient en majorité des hommes (57 %) avec un âge moyen de 38,7 ans, et 93 % provenaient de Bamako. Les signes cliniques les plus fréquents étaient les douleurs musculaires et articulaires (87,7 %) et la fièvre (83,8 %). Selon la classification de l’OMS, plus de 44 % des cas étaient classés B ou C (formes modérées à graves), avec une fréquence élevée de complications biologiques : cytolyse hépatique (47,3 %), thrombopénie sévère (28,0 %), leucopénie (24,5 %), et insuffisance rénale (12 %). Les formes hémorragiques représentaient 43,7 % des cas graves. La durée moyenne d’hospitalisation était de 6,7 ± 6,2 jours. La mortalité hospitalière était particulièrement préoccupante, atteignant 26,1 %, un chiffre bien supérieur aux taux habituellement rapportés. Cette surmortalité pourrait s’expliquer par le retard au diagnostic, l’automédication préalable (traitement antipaludique injustifié dans plus de 65 % des cas), ou encore des défaillances d’accès à la réanimation. Cette étude est un signal d’alerte fort sur la virulence de la dengue dans le contexte malien, et souligne la nécessité de renforcer les capacités de diagnostic, de surveillance et de prise en charge, tout en sensibilisant les professionnels de santé aux formes atypiques de fièvre dans les zones non palustres.

# Research article: Influence des variations climatiques sur l’incidence de la dengue au Sénégal de 2022 à 2024

**Published:** 2026-01-23

**Authors:** Crédo HOUNDODJA

**Affiliations:** Solthis, Dakar, Sénégal

La dengue représente un défi majeur de santé publique au Sénégal, avec une augmentation significative des cas ces dernières années. Le changement climatique, à travers ses effets sur la température et les précipitations, modifie les dynamiques de transmission des arboviroses en influençant la prolifération des moustiques vecteurs. Dans le contexte sénégalais, caractérisé par sa diversité climatique et écologique, une meilleure compréhension de ces relations est essentielle pour guider les politiques de santé publique. Cette étude vise à analyser l’influence des variations climatiques sur l’incidence de la dengue au Sénégal entre 2022 et 2024, à identifier les zones géographiques les plus touchées, et à prédire l’évolution future des cas pour orienter les stratégies de prévention et de contrôle. Cette étude descriptive rétrospective et analytique a utilisé les données épidémiologiques de

la dengue recueillies par la Direction de surveillance épidémiologique du ministère de la Santé du Sénégal de janvier 2022 à septembre 2024. Les données climatiques (température et précipitations) ont été obtenues à partir de sources satellitaires. Une analyse géospatiale a été réalisée pour déterminer la distribution régionale des cas, tandis que des analyses de corrélation ont été effectuées entre l’incidence de la dengue et les variables climatiques. Le modèle Prophet a été utilisé pour modéliser et prédire l’évolution des cas jusqu’en 2025. L’analyse géospatiale a révélé une distribution hétérogène des cas de dengue, avec une concentration dans les régions de Dakar (293 cas) et Matam (195 cas), suivies de Thiès (56 cas) et Saint Louis (32 cas). L’évolution annuelle montre une augmentation significative à Dakar en 2023 (212 cas contre 48 en 2022), suivie d’une diminution en 2024 (32 cas jusqu’en septembre). Une corrélation significative a été observée entre la température et l’incidence de la dengue, avec une transmission optimale entre 25°C et 29°C. Une diminution notable des cas a été constatée lorsque les températures dépassaient 30°C, illustrant l’effet limitant des températures extrêmes sur la survie des moustiques vecteurs. Concernant les précipitations, un décalage temporel a été identifié : les pics d’incidence survenaient principalement entre octobre et décembre, soit 1 à 2 mois après les pics de précipitations (août-septembre). La modélisation temporelle a mis en évidence une saisonnalité marquée et prévoit un pic attendu pour le quatrième trimestre 2025, permettant d’anticiper les périodes à risque. Cette étude démontre l’influence significative des variations climatiques sur la transmission de la dengue au Sénégal. Les températures optimales (25-29°C) favorisent la prolifération des moustiques vecteurs, tandis que le décalage observé entre les précipitations et les pics d’incidence souligne l’importance de cibler les interventions préventives durant la période post-pluies. Les disparités régionales observées appellent à des stratégies différenciées selon les contextes écologiques et urbains. Ces résultats fournissent des informations essentielles pour guider les stratégies de surveillance et de lutte antivectorielle, particulièrement dans les zones urbaines comme Dakar et les régions rurales à proximité de plans d’eau comme Matam.

# Research article: Analyse de la distribution spatiale des cas de fièvre hémorragique de Crimée-Congo (FHCC) et facteurs associés à sa mortalité au Sénégal

**Published:** 2026-01-23

**Authors:** Mayassine DIONGUE

**Affiliations:** Institut de santé et développement (ISED), Université Cheikh Anta Dakar, Sénégal

La fièvre hémorragique de Crimée-Congo (FHCC) est une arbovirose grave transmise principalement par les tiques du genre *Hyalomma*, avec un taux de létalité pouvant atteindre 40 %. Elle constitue une menace zoonotique émergente en Afrique de l’Ouest. Au Sénégal, une séroprévalence de 1,8 % a récemment été rapportée, notamment dans les régions nord et centre. Les changements climatiques, les pratiques d’élevage extensif et les mobilités humaines contribuent à l’extension du risque de transmission. Cette étude vise à décrire les caractéristiques épidémiologiques des cas de FHCC notifiés au Sénégal entre janvier 2023 et septembre 2024, à identifier les facteurs associés à la mortalité et à évaluer l’efficacité des systèmes de détection en place. Il s’agit d’une étude rétrospective descriptive basée sur les données collectées par la Direction de la surveillance épidémiologique du ministère de la Santé du Sénégal. Une analyse géospatiale a été réalisée pour cartographier la distribution des cas. Les variables cliniques, biologiques et démographiques ont été analysées afin d’identifier les facteurs de risque de décès et d’évaluer la performance des outils de surveillance. Dix-huit cas confirmés ont été enregistrés sur la période, avec une prédominance masculine (72,2 %) et un âge médian de 34,5 ans. La majorité des cas provenait de Dakar (33,3 %), suivie de Matam (16,7 %), puis de Fatick, Louga et Kaolack (11,1 % chacune). Le taux de létalité global était de 22,2 %, les quatre décès étant tous associés à des signes hémorragiques sévères. Ces manifestations, observées chez 44,4 % des patients, apparaissent comme un facteur de risque majeur de mortalité. Le Réseau 4S a permis la détection de 50 % des cas, démontrant son importance dans la surveillance nationale, tandis que 38,9 % des cas ont été identifiés à travers les investigations de fièvres hémorragiques virales. Le diagnostic était principalement basé sur la PCR combinée à la détection des IgM (55,6 %). Un cas importé de Mauritanie a été rapporté, mettant en lumière le risque de circulation transfrontalière. Les cas sont survenus de manière continue tout au long de l’année, sans pic saisonnier marqué. Ces résultats soulignent l’urgence de renforcer les capacités de surveillance, de diagnostic et de prise en charge précoce, ainsi que la formation du personnel de santé et la sensibilisation communautaire pour limiter l’impact de cette maladie potentiellement létale.

# Research article: Létalité et facteurs associés à la survenue du décès au cours du paludisme grave au Service des maladies infectieuses et tropicales du CHNU Fann à Dakar

**Published:** 2026-01-23

**Authors:** Mamadou Lamine DIA

**Affiliations:** Service des maladies infectieuses, Centre hospitalier national universitaire de Fann, Dakar, Sénégal

Le paludisme grave constitue toujours une cause majeure de mortalité dans les pays d’endémie, en particulier en Afrique subsaharienne. L’objectif de cette étude était d’identifier les facteurs associés à la survenue du décès au cours du paludisme grave. Il s’agissait d’une étude rétrospective, descriptive et analytique sur une période de 6 ans, du 1^er^ janvier 2018 au 31 décembre 2023 portant sur des patients hospitalisés pour paludisme grave au Service des maladies infectieuses et tropicales (SMIT) du CHNU de Fann. Le diagnostic de paludisme grave a été retenu conformément aux critères de l’OMS de 2015. Un total de 375 cas avait été colligé, soit une fréquence hospitalière de 8,95 %. L’âge médian était de 29 ans (IQR : 21,47), le sexratio (H/F) de 3,12. Le principal signe clinique de gravité était la prostration (64,8 %) suivie de l’ictère (41,6 %) et du coma (40,5 %). Les critères paracliniques les plus observés étaient l’insuffisance rénale (17,1 %), l’anémie (10,7 %). Une hyperparasitémie était notée dans 2,7 % des cas. Plus de la moitié des patients présentait chacun au moins deux critères de gravité du paludisme. Le traitement reposait sur l’administration de l’artésunate (92,8 %). La létalité se chiffrait à 13,87 %. Les facteurs associés à la survenue du décès étaient l’infection à VIH (ORa=10,1; IC95 % : 2,10–49,1; p<0,01), l’OAP (ORa=4,17; IC95 % : 1,18–14,3; p<0,05), l’insuffisance rénale (ORa=4,03; IC95 % : 1,59–10,4; p<0,01), l’association d’une méningite bactérienne (ORa=3,14; IC95 % : 1,12–8,26; p<0,05), la survenue de complications (ORa=10,6; IC95 % : 2,95–56,5; p=0,001) et le nombre de doses d’artésunate ≥3 (ORa=0,02; IC95 % : 0,01–0,06; p<0,001). La létalité du paludisme grave est encore accrochée au SMIT de Fann. Une survenue accrue devrait être de mise chez les PVVIH, la présence d’une insuffisance rénale et rechercher une méningite associée au paludisme notamment en cas de persistance de la fièvre.

# Research article: Brucellose bovine en Afrique de l’Ouest et du Centre : prévalence et enquêtes CAP dans les exploitations laitières périurbaines

**Published:** 2026-01-23

**Authors:** Andrée Prisca Ndjoug NDOUR

**Affiliations:** École inter-États des sciences et médecine vétérinaires de Dakar (EISMV), Université Cheikh Anta Diop de Dakar, Sénégal

Cette présentation rend compte d’une vaste enquête séro-épidémiologique et socio-comportementale menée entre 2017 et 2020 dans les exploitations laitières périurbaines de plusieurs pays d’Afrique de l’Ouest et du Centre. Le projet a évalué la prévalence de la brucellose bovine dans les zones périurbaines laitières, à travers deux tests (iELISA sur lait de mélange et Rose Bengale sur sérums) sur 1 864échantillons de lait et 3 963 sérums. Parmi les laits de mélange testés, la prévalence globale était de 12 % (15,9 % en Afrique de l’Ouest, 11,1 % en Afrique centrale) et parmi les sérums la prévalence globale était de 10 % (9,5 % en Afrique de l’Ouest et 12,5 % en Afrique centrale). On retrouvait une forte hétérogénéité entre pays : prévalence alarmante au Togo (62 % Elisa, 29,5 % Rose Bengale), mais très faible au Sénégal (<0,5 % quel que soit le test). L’enquête CAP (connaissances, attitudes, pratiques) montrait un déficit important d’information chez les éleveurs. Ainsi, 46 % et 73 % d’entre eux, en Afrique centrale et de l’Ouest, respectivement, n’avaient jamais entendu parler de la brucellose et seulement 7 % comprenaient les modes de transmission. Pratiquement aucun n’identifiait la maladie comme zoonotique. Cette étude met en évidence le caractère silencieux mais préoccupant de la brucellose dans les chaînes de production laitière, et souligne la nécessité d’une approche holistique prenant en compte les relations entre l’humain, l’animal et l’environnement. Il est nécessaire d’insister sur la sensibilisation des éleveurs, la surveillance vétérinaire et la collaboration intersectorielle.

# Research article: Séroprévalence de la fièvre de la Vallée du Rift chez les ruminants domestiques de la région de Tahoua (Niger)

**Published:** 2026-01-23

**Authors:** Souahibou SOUROKOU SABI

**Affiliations:** École inter-États des sciences et médecine vétérinaires de Dakar (EISMV), Université Cheikh Anta Diop de Dakar, Sénégal

Cette étude conduite en 2024 dans la région de Tahoua, zone pastorale endémique, visait à évaluer la circulation virale de la fièvre de la Vallée du Rift (FVR) chez les ruminants domestiques. Un total de 615 échantillons sanguins a été collecté chez des bovins, ovins, caprins et camelins dans 22 communes de la région de Tahoua qui est une zone endémique de la FVR. Les tests ELISA ont révélé une prévalence globale de 11,9 %, avec des taux particulièrement élevés chez les camelins (36,5 %) et les bovins (17,7 %). En termes d’âge, 12,3 % des adultes sont infectés contre 9,8 % des jeunes, sans différence significative entre les femelles et les mâles. Cette prévalence, bien plus importante que dans les données historiques nigériennes (3 à 7 % hors période d’épidémie mais 20 % en période post-épidémie en 2016-2018), est attribuée à des facteurs tels que les mouvements transfrontaliers du bétail pour le commerce et la recherche de pâturage en périodes de soudure. Les auteurs insistent sur la nécessité de renforcer la surveillance vétérinaire et les recherches épidémiologiques.

# Research article: Leptospirose chez les petits ruminants et les chevaux à Dakar

**Published:** 2026-01-23

**Authors:** Mireille Souahibou SOUROKOU SABI

**Affiliations:** École inter-États des sciences et médecine vétérinaires de Dakar (EISMV), Université Cheikh Anta Diop de Dakar, Sénégal

La leptospirose est une zoonose négligée mais potentiellement grave. Cette enquête sérologique menée de mai à juillet 2023 a ciblé les chevaux (n=90), ovins (n=61) et caprins (n=27) des zones urbaine et périurbaine de Dakar. Les échantillons sanguins (n=178) ont été analysés par MAT au laboratoire de la faculté de médecine vétérinaire de l’Université de Zagreb (Croatie). Ils ont révélé une séroprévalence globale de 9,0 %. Les chevaux étaient les plus atteints (17,8 %), tandis que les ovins (4,9 %) et caprins (0 %) étaient faiblement ou pas du tout positifs. Plusieurs sérovars pathogènes ont été identifiés, notamment *Australis*, *Canicola, Icterohaemorrhagiae* et *Tarassovi*. L’étude souligne l’importance du rôle des chevaux dans la transmission potentielle à l’humain, surtout dans un environnement urbain où la promiscuité humain-animal est élevée. Les auteurs appellent à un renforcement de la surveillance et à l’élaboration de stratégies de sensibilisation pour un meilleur contrôle de cette zoonose.

# Research article: Rage humaine au Sénégal : état des lieux, défis et perspectives

**Published:** 2026-01-23

**Authors:** Aboubakar Sidikh BADIANE

**Affiliations:** Service des maladies infectieuses et tropicales du CHNU de Fann, Dakar, Sénégal

Cette communication conjointe entre le SMIT du CHNU de Fann et l’Institut Pasteur de Dakar a dressé un tableau complet de la situation de la rage au Sénégal. Malgré la gravité de la maladie et son caractère évitable, la rage reste sous-diagnostiquée et sous-déclarée, avec des foyers actifs dans 9 régions du pays. Entre 1986 et 2017, 97 cas humains ont été notifiés au Sénégal. Entre 2013 et 2023, plus de 34 000 cas d’exposition humaine à la rage ont été rapportés, dont une grande majorité par morsure de chien. En 2020, des foyers de rage animale ont été identifiés dans 9 des 14 régions du Sénégal. Les professionnels essaient de remplacer progressivement le protocole intramusculaire comprenant 3 visites par le protocole intradermique à 2 visites. Entre 2018 et 2023, 1 351 patients ont été reçus au SMIT du CHNU de Fann, centre de traitement antirabique du Sénégal, pour prophylaxie post-exposition (PPE), mais la moitié n’a pas complété le schéma vaccinal (47 % 1 seule dose). Les principaux freins sont l’accessibilité des vaccins, le coût (le patient doit payer lui-même ses traitements), le manque d’information et l’insuffisance de formation du personnel. Les facteurs de risque associés à un retard de la PPE sont une résidence hors de Dakar et une morsure par un animal non agressif. Les orateurs ont souligné la nécessité d’une approche coordonnée et multisectorielle pour la lutte contre la rage, une intégration des activités de contrôle de la rage avec celles des autres MTN, une amélioration de la disponibilité des vaccins pour les humains et les animaux, et d’une augmentation des capacités diagnostiques au Sénégal. Des financements supplémentaires sont nécessaires pour généraliser la vaccination et améliorer l’approvisionnement national. Enfin, une étude pilote de surveillance intégrée au Sénégal *via* l’application smartphone REACT a été présentée, permettant d’identifier les différentes

étapes de la gestion intégrée des cas de morsures : notification, investigation, quarantaine, laboratoire, déclaration. Ils ont enfin présenté des méthodes diagnostiques innovantes et facilement transportables « Laboratoire dans une valise », utilisable pour le diagnostic *ante-mortem* de la rage chez l’humain et pour la confirmation de la rage animale dans le secteur vétérinaire et dans les zones reculées.

# Research article: Bilan de prise en charge des cas de morsures à l’unité de vaccination du SMIT de FANN

**Published:** 2026-01-23

**Authors:** Aïssa SOW

**Affiliations:** Service des maladies infectieuses et tropicales du CHNU de Fann, Dakar, Sénégal

La rage est une zoonose accidentellement humaine, qui provoque une encéphalomyélite aiguë mortelle, due à un virus du genre *Lyssavirus* de la famille des Rhabdovirus. Elle est transmise par la bave infectée de divers animaux, après griffure, léchage ou morsure. La rage est mortelle à 100 % en l’absence de prophylaxie. Au Sénégal, la notification des cas de morsure fait partie des indicateurs sous surveillance. L’objectif de notre étude était de décrire les caractéristiques épidémiologiques et cliniques et leur prise en charge au sein de l’unité de vaccination du Service des maladies infectieuses et tropicales (SMIT) du CHNU de Fann à Dakar. Cette étude transversale rétrospective a été réalisée dans ce service du 1^er^ janvier et au 31 décembre 2024. Les données ont été recueillies à partir de l’unité de vaccination. Les informations de la fiche de notification des cas de morsures ont été exploitées et renseignées au niveau d’une fiche *google form*. Les données ont été analysées grâce au logiciel Epi info. Au total, 474 cas de morsures ont été pris en charge au sein de l’unité de vaccination du SMIT de Fann en 2024. L’âge moyen des patients était de 27 ans. Trente-neuf pour cent des patients avaient moins de 18 ans. Il y avait une nette prédominance masculine (sex-ratio : 2,18). Dans la majorité des cas (98,7%), les patients résidaient dans la région de Dakar. L’animal mordeur était principalement le chien (86,7 %) suivi du chat (11 %). Plus de la moitié des cas (55 %) a été prise en charge entre les mois d’août et de novembre. Il s’agissait d’une morsure dans 439 cas (92,6 %). Les sièges de morsure ou griffures les plus fréquents étaient la jambe (30 %), la main (17 %) et la cuisse (13 %). L’évaluation de la gravité retrouve que 93 % des expositions étaient classées en catégorie 3. Concernant l’animal mordeur, il était agressif dans 45 % des cas. Il était soit non vacciné (45,6 %), soit avec un statut vaccinal inconnu (41 %). La victime ne connaissait pas le devenir de l’animal dans plus de la moitié des cas (55 %). Immédiatement après la morsure, la plaie n’avait pas été lavée dans 51 % des cas. Les cas d’exposition au risque rabique restent relativement fréquents. L’animal mordeur est le chien errant et la prise en charge initiale est inadéquate. Des actions doivent être menées par autorités afin de réduire le nombre d’expositions mais aussi afin de sensibiliser la population sur l’importance de prophylaxie post exposition après griffure, léchage ou morsure.

**Figure 2:** Photos prises lors du 2^e^ congrès de la Société sénégalaise de pathologie infectieuse et tropicale (SOSEPIT), Dakar 28 au 30 avril 2025 (crédit photo : L. Fortes et L. Epelboin)

# Research article: À la fin du congrès, des recommandations ont été formulées sur différentes thématiques de l’infectiologie

**Published:** 2026-01-23

**La résistance aux antimicrobiens**

- Mettre en place des équipes mobiles d
- ’antibiothérapie dans les structures sanitaires pour un suivi optimal des prescriptions.
- Améliorer la régulation et le contrôle de l’utilisation des antibiotiques.
- Soutenir la recherche locale pour développer des alternatives thérapeutiques aux antibiotiques.
- Renforcer les capacités des laboratoires (TDR, techniques et phénotype de résistance).
- Opérationnaliser les instances « *One Health* » à tous les niveaux du système de santé.
- Encourager la recherche multisectorielle sur la résistance aux antimicrobiens (RAM) et les alternatives aux antibiotiques (phytothérapie).

**La prévention et le contrôle de l’infection**

- Redynamiser les comités de lutte contre les infections nosocomiales.
- Respecter les mesures d’asepsie lors de la mise en place des dispositifs médicaux.
- Renforcer la surveillance des infections associées aux soins.
- Former le personnel de santé à la PCI.
- Réaliser des audits des pratiques.
- Élaborer et diffuser des protocoles standardisés de PCI.

**Les maladies tropicales négligées**

- Renforcer la recherche, l’innovation et l’adoption de l’approche « *One Health* », afin de mieux comprendre les dynamiques des MTN et d’améliorer la réponse multisectorielle.
- Promouvoir une approche intégrée et multisectorielle, en assurant l’alignement des interventions de lutte contre les MTN avec les politiques et stratégies nationales de santé publique.
- Soutenir le développement et l’accès à des outils de diagnostic rapides, sensibles et adaptés, afin d’accélérer l’identification des cas, réduire la morbidité et progresser vers l’élimination des MTN.
- Exploiter le potentiel des technologies numériques et de l’intelligence artificielle, en développant de nouveaux outils de surveillance, de diagnostic et de prise en charge, pour renforcer les capacités locales et améliorer l’efficacité des interventions.

**Les fièvres hémorragiques virales et autres maladies émergentes**

- Renforcer les capacités diagnostiques à l’échelle régionale, en dotant les structures sanitaires de plateformes de biologie moléculaire et en assurant leur maintenance.
- Mettre en place des Centres de traitement des épidémies (CTE) conformes aux normes internationales, dans les régions à risque, pour une prise en charge rapide et sécurisée des cas.
- Diffuser les procédures opérationnelles standardisées (SOP) pour la détection, la notification, la référence et la gestion des cas de FHV.

**Le diagnostic et la prise en charge des infections fongiques**

- Développer la formation et la recherche appliquée, en intégrant les infections fongiques dans les curricula universitaires et en soutenant les projets locaux de recherche.
- Améliorer les capacités des laboratoires, en dotant les structures de santé de moyens techniques pour le diagnostic mycologique (culture, immunodiagnostic, biologie moléculaire).
- Assurer la disponibilité des antifongiques essentiels, notamment les traitements de première ligne comme l’ampho-téricine B, le fluconazole, le voriconazole et les échinocandines.
